# Comprehensive Analysis of circRNA Expression Profiles During Cervical Carcinogenesis

**DOI:** 10.3389/fonc.2021.676609

**Published:** 2021-08-31

**Authors:** Haixia Luo, Yuanxing Li, Yueyang Zhao, Jingjing Chang, Xiu Zhang, Binbin Zou, Lifang Gao, Wei Wang

**Affiliations:** ^1^Department of Obstetrics and Gynecology, The Second Hospital of Shanxi Medical University, Taiyuan, China; ^2^Department of Pathology & Shanxi Key Laboratory of Carcinogenesis and Translational Research on Esophageal Cancer, Shanxi Medical University, Taiyuan, China; ^3^Department of Pathology, The Second Hospital of Shanxi Medical University, Taiyuan, China

**Keywords:** cervical cancer, high-grade squamous intraepithelial lesions (HSIL), cervical intraepithelial neoplasia (CIN), circRNAs, cell cycle, bioinformatics

## Abstract

Circular RNAs (circRNAs) are regulatory molecules that participate in the occurrence, development and progression of tumors. To obtain a complete blueprint of cervical carcinogenesis, we analyzed the temporal transcriptomic landscapes of mRNAs and circRNAs. Microarrays were performed to identify the circRNA and mRNA expression profiles of cervical squamous cell carcinoma (CSCC) and high-grade squamous intraepithelial lesion (HSIL) patients compared with normal controls (NC). Short time-series expression miner (STEM) was utilized to characterize the time-course expression patterns of circRNAs and mRNAs from NC to HSIL and CSCC. A total of 3 circRNA profiles and 3 mRNA profiles with continuous upregulated patterns were identified and selected for further analysis. Furthermore, functional annotation showed that the mRNAs were associated with DNA repair and cell division. The protein-protein interaction (PPI) network analysis revealed that the ten highest-degree genes were considered to be hub genes. Subsequently, a competing endogenous RNA (ceRNA) network analysis and real-time PCR validation indicated that hsa_circ_0001955/hsa-miR-6719-3p/CDK1, hsa_circ_0001955/hsa-miR-1277-5p/NEDD4L and hsa_circ_0003954/hsa-miR-15a-3p/SYCP2 were highly correlated with cervical carcinogenesis. Silencing of hsa_circ_0003954 inhibited SiHa cell proliferation and perturb the cell cycle *in vitro*. This study provides insight into the molecular events regulating cervical carcinogenesis, identifies functional circRNAs in CSCC, and improves the understanding of the pathogenesis and molecular biomarkers of CSCC and HSIL.

## Introduction

Cervical cancer is one of the most prevalent gynecological malignancies and the fourth most fatal cancer (1). Cervical squamous cell carcinoma (CSCC) is the most frequent pathological subtype of cervical cancer, accounting for approximately 80-85% of cases (2). Uterine cervix carcinogenesis is a step-by-step process that shows a continuum of neoplastic transitions from the persistence of high-risk human papillomavirus (HR-HPV, mostly HPV16) infection to low-grade squamous intraepithelial lesion (LSIL) to high-grade squamous intraepithelial lesion (HSIL) and then to invasive cancer histologically (3). It has been reported that almost half of HSILs will progress to invasive cancer (4, 5). However, the molecular mechanism of cervical carcinogenesis is not completely understood, and previous studies of cervical precancerosis have been extremely limited. Hence, exploration of the genetic changes and epigenetic modifications may reveal new clues to elucidate the molecular mechanisms of cervical carcinogenesis.

Circular RNAs (circRNAs), a kind of noncoding RNA without a 3′ tail or 5′ cap, are naturally occurring endogenous molecules (6, 7). The microRNA (miRNA) response elements (MREs) in circRNAs can suppress the function of miRNAs, during this process, circRNAs inhibit the activity of miRNAs and modulate the expression of their downstream target genes in various types of malignancies (8). Previous research has highlighted the important roles of circRNAs in cancer occurrence and progression (9–14). Recently, multiple studies have identified the participation of circRNAs in CSCC formation and progression (15, 16). Cervical cancer tissue samples and cell lines both highly expressed CiRS-7, and its expression levels correlated significantly with prognosis (17). CircE7 in CaSki cervical carcinoma cells inhibits the growth of cancer cells by reducing the protein levels of E7 (18). However, while the majority of research has primarily focused on identifying the differentially expressed circRNAs (DECs) between cervical cancer and controls, few researchers have focused on HSIL. The role of DECs in cervical carcinogenesis has not been systematically described.

In this study, we recruited not only CSCC patients and controls but also HSIL individuals. Due to the stepwise progression of CSCC, the expression of circRNAs and mRNAs could be dysregulated at any specific tumorigenesis stage. To characterize the changes in circRNA and mRNA expression, we performed trend analysis to identify the predominant circRNAs and mRNAs in the control, HSIL and CSCC groups. This study provides a starting point for further research into the molecular mechanism of circRNAs in cervical carcinogenesis, which provides new insight into the multiple and complex factors in early cervical carcinogenesis.

## Materials and Methods

### Patients and Specimens

A total of 35 human cervical specimens were acquired, including 12 patients with only HPV16-positive normal controls (NC), 11 with only HPV16-positive HSIL and 12 with only HPV16-positive stage IA-IIA CSCC, who were collected at the Second Hospital of Shanxi Medical University between December 2019 and October 2020. We collected all patients’ clinical information, including age, HPV testing, the ThinPrep^®^ Pap Test (TCT) and pathology (by colposcopy biopsy, cervical conization or hysterectomy) results. Based on the International Federation of Gynecology and Obstetrics (FIGO) criteria, the clinical stage of the patients with CSCC was determined (19). The diagnosis of all of the cases was histologically confirmed by two independent pathologists, and all of the CSCC (**Figures S1A–C** **)** and HSIL (**Figures S1D–F**) samples were assessed by hematoxylin and eosin (H&E) staining. Cases were excluded when they met any of the following exclusion criteria: 1) had a history of cervical physical therapy (ablation and cryosurgery), chemotherapy and pelvic radiotherapy; 2) had other tumors; and 3) had an immunocompromised condition (e.g., infection with human immunodeficiency virus). The clinicopathological characteristics of the patients and the usage of specimens are summarized in **Table S1**. This study was approved by the Ethics Committees of the Second Hospital of Shanxi Medical University.

### Cell Culture

SiHa (human cervical squamous cell carcinoma cell line, HPV 16-positive) and HcerEpic (human normal cervical epithelial cell line) cells (ATCC, Rockville, MD, USA) were cultured with DMEM (General Electric Company, USA) with 10% fetal bovine serum (Gibco; Thermo Fisher Scientific, Inc., Waltham, MA, USA), 100 mg/mL streptomycin, and 100 U/mL penicillin in a humidified atmosphere of 5% CO_2_ and 37°C.

### RNA Extraction and Quality Control

Total RNA was extracted from tissues using TRIzol Reagent (Life Technologies, CA, US) and purified with an RNeasy Mini Kit (Qiagen, Valencia, CA, USA). Assaying the purity and integrity of the RNA was achieved using agarose gel electrophoresis and a UV/vis spectrophotometer (Thermo, NanoDrop 2000, USA).

### Microarray Hybridization and Data Analysis

Microarray hybridization was performed in accordance with the Affymetrix GeneChip Expression Analysis Technical Manual (www.affymetrix.com). The data were analyzed using the robust multichip analysis (RMA) algorithm using default Affymetrix settings. The values presented are the log2 RMA signal intensity. Differentially expressed mRNAs (DEMs) and DECs among the three groups were filtered according to the following criteria: *p* < 0.05 and fold change > 1.2. The microarray data were uploaded to a public database (accession number: GES166466).

### Functional Enrichment Analysis

The online tool Metascape (http://metascape.org/) was used to analyze DEMs (20). Three types of gene ontology (GO) were used to functionally enhance biological process (BP), molecular function (MF) and cellular component (CC) (21). Kyoto Encyclopedia of Genes and Genomes (KEGG) pathway enrichment was also performed.

### Short Time-Series Expression Miner (STEM)

STEM software version 1.3.13 (http://www.cs.cmu.edu/~jernst/stem) was utilized to cluster and view probable circRNA and mRNA expression patterns over time (22). The STEM clustering method and other options were set as defaults. The circRNA and mRNA expression profiles were clustered based on statistically significant values (*p *< 0.05).

### Protein-Protein Interaction (PPI) Network Construction

The PPI network of DEMs was constructed using the STRING (a search tool for the retrieval of interacting genes, http://string-db.org) online database (23). In the current study, a combined score ≥ 0.99 was considered the cutoff criterion. The PPI network was visualized on a free bioinformatics platform provided by Cytoscape version 3.7.1 (https://cytoscape.org/). MCODE (molecular complex detection) version 3.7.1 The Cytoscape plugin was used to screen the potential hub modules.

### Competing Endogenous RNA (ceRNA) Network Construction

CircRNA–miRNA interactions were predicted using the miRanda (http://www.microrna.org/microrna/home.do) online database (24), and miRNA–mRNA interactions were predicted using the TargetScan (http://www.targetscan.org/vert_72/) (25) and miRanda prediction databases. The ceRNA networks were visualized by Cytoscape.

### qRT-PCR and CSCD (Cancer-Specific CircRNA Database) Analysis

Quantitative real-time polymerase chain reaction (qRT-PCR) was performed using Q SYBR Green Supermix (Bio-Rad, Hercules, CA, USA), and PCR-specific amplification was conducted in the 7900 HT Sequence Detection System (ABI PRISM; Waltham, MA, USA). The expression was determined by using the threshold cycle (Ct) method, and relative expression levels were calculated *via* the 2^–ΔΔCt^ method. The top five circRNAs with the highest degree in the ceRNA network and the top five miRNAs and mRNAs for the potential circRNAs were validated by qRT-PCR. The primers for these RNAs are presented in **Table S2**. CSCD (http://gb.whu.edu.cn/CSCD/) (26), an online tool to investigate cancer-specific circRNAs, was utilized to obtain the structure of potential circRNAs.

### Transfection

To transfect with siRNA, we used custom-designed siRNAs targeting hsa_circ_0003954 (**Table S3** and **Figure 10B**). For transfection, SiHa cells were grown on 6 well plates. Cells were transfected at 24 h with 30 pmol siRNA or scrambled control (GenePharma, Shanghai, China) using Lipofectamine 3000 (Invitrogen MA, USA) according to the manufacturer’s protocol. A total of three biological triplicates were conducted.

### Cell Proliferation and Cell Cycle Analysis

Cell proliferation was detected through the CCK-8 assay (Meilunbio, Dalian, China). For transient transfection experiments, 1×10^3^ cells were plated in 96 well plates for 24 hours at 37°C. Proliferation absorbance was measured with a multifunctional microplate reader (SpectraMax M5, MD, USA). Experiments were repeated three times. Cell cycle assays were conducted using propidium iodide stained SiHa cells by a Beckman Coulter FC500 flow cytometer (Beckman-Coulter, Hialeah, FL) and analyzed using Modfit software.

### Statistical Analysis

Experimental data are presented as the mean ± standard deviation (SD) of at least three experiments. Significant differences were assessed by Student’s t-test. *P* < 0.05 was considered statistically significant. Figures were drawn using R Studio version 3.3.4 (The R Foundation for statistical computing, Vienna, Austria).

## Results

### Analysis of circRNA and mRNA Expression Profiles

**Figure 1:**
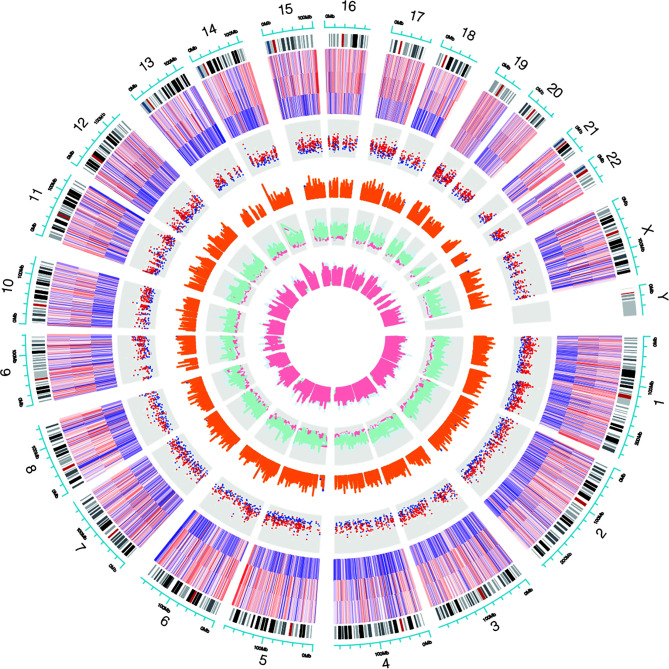
Chromosomal distribution of the candidate circRNAs. Chromosomes 1, 2, 3 and X contained more circRNAs than the other chromosomes.

**Figure 2:**
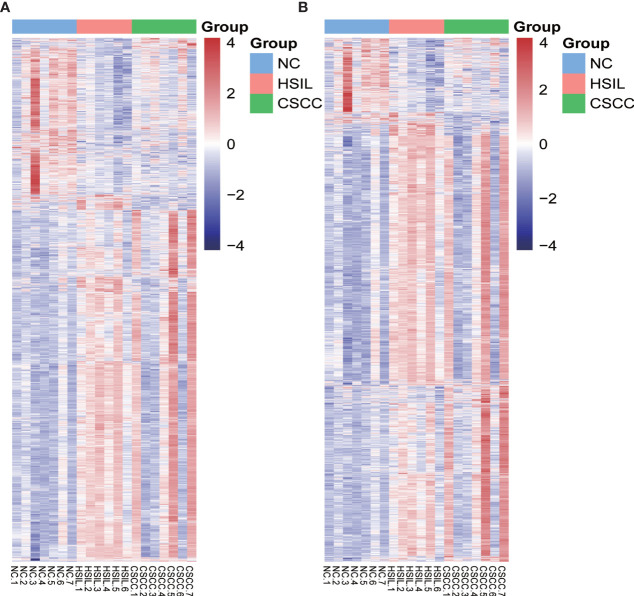
Heatmap of differentially expressed mRNAs (DEMs) and circRNAs (DECs) in the three comparison groups. Genes and samples (rows and columns, respectively) are reordered on the basis of the normalized expression value and give rise to groups of samples and genes with similar expression patterns, according to the color key. The samples (column) were clustered into three groups according to cervical sample: seven individuals with NC, six individuals with HISL and seven individuals with CSCC. **(A)** DEMs; **(B)** DECs.

Genome-wide analysis of differentially expressed profiles in circRNA and mRNA among CSCC, HSIL and NC tissues was performed. A total of 3172 circRNA candidates were differentially expressed with a fold change ≥ 1.2 (*p* < 0.05). Thirty-two circRNAs revealed a fold change ≥ 10. Hsa_circ_0066984 (fold change ~ 24) was the most dysregulated circRNA. The candidate DECs were distributed on 46 human chromosomes, including the chromosomes 1, 2, 3 and X chromosome, which contained more circRNAs than the other chromosomes (**Figure 1**). A total of 4519 mRNAs among CSCC, HSIL and NC tissues had a fold change ≥ 1.2 (*p* < 0.05). Summarization of the coding gene expression profile showed that 46 mRNAs displayed a fold change ≥ 10. CircRNA and mRNA expression patterns among CSCC, HSIL and NC samples were significantly differentially expressed, as shown by hierarchical clustering (**Figure 2**). The cluster heatmaps of the DECs and DEMs showed good discrimination among CSCC, HSIL and NC samples.

### Functional Annotation of DEMs

**Figure 3:**
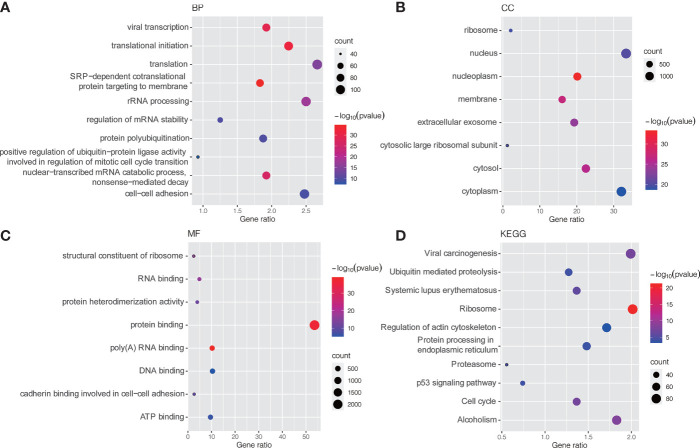
GO and KEGG pathway enrichment analysis of DEMs. **(A)** Bubble plot of BP; **(B)** Bubble plot of CC; **(C)** Bubble plot of MF; **(D)** Bubble plot of KEGG. GO, Gene Ontology; KEGG, Kyoto Encyclopedia of Genes and Genomes; BP, biological process; CC, cellular component; MF, molecular function.

GO and KEGG pathway enrichment analyses were conducted to explore potential biological processes and pathways enriched by DEMs. **Figure 3** presents the top ten enriched BP, CC, MF terms and KEGG pathways. The enriched BP terms were mainly related to carcinogenic processes, such as viral transcription, translational initiation, regulation of mRNA stability and positive regulation of ubiquitin−protein ligase activity involved in regulation of mitotic cell cycle transition (**Figure 3A** **),** the most enriched CC was the nucleus (**Figure 3B**) and the most enriched MF was protein binding (**Figure 3C**). Similar to the GO term results, KEGG pathway enrichment analysis found that DEMs were mainly enriched in viral carcinogenesis, ubiquitin-mediated proteolysis, protein processing in endoplasmic reticulum, p53 signaling pathway and cell cycle (**Figure 3D** **)**.

### Temporal Gene Expression Patterns of mRNAs and circRNAs

**Figure 4:**
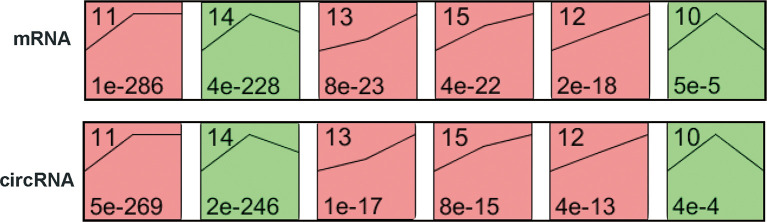
STEM identified the temporal expression profiles of mRNAs and circRNAs with *p* < 0.05. The black lines in the profile boxes depict the gene expression patterns over the three time points. The profile number on the top left corner of each profile box was assigned by STEM, and the number on the bottom left represents the adjusted *p*-value. Profiles 12, 13 and 15 had the same continuous upregulation patterns. STEM, short time-series expression miner.

**Figure 5:**
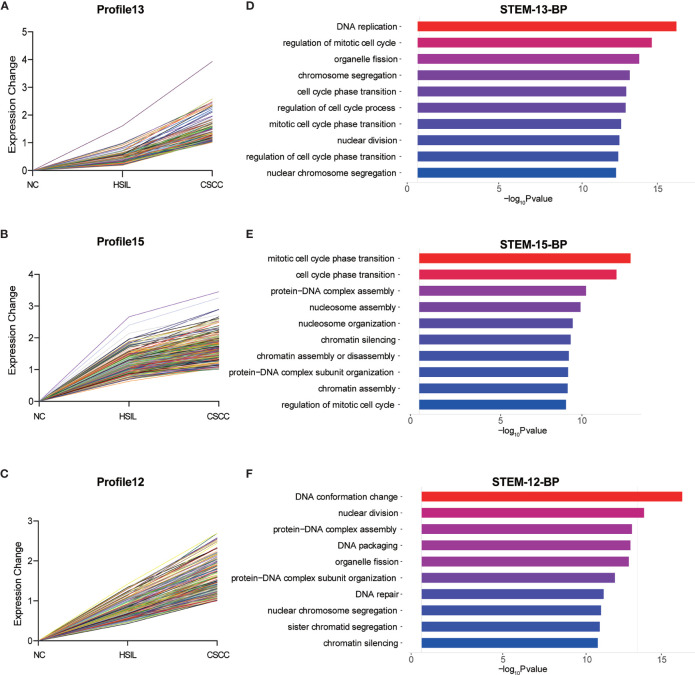
Expression patterns of profiles 13 **(A)**, 15 **(B)** and 12 **(C)** and GO analysis of genes clustered in 13 **(D)**, 15 **(E)** and 12 **(F)**. GO, Gene Ontology; BP, biological process; STEM, short time-series expression miner.

To explore the differences in gene expression between cervical carcinogenesis stages, the STEM tool was applied to profile stage-specific gene expression patterns. The microarray data were normalized to the NC data, and the temporal gene expression profiles were identified. Six temporal mRNA profiles and six temporal circRNA profiles were statistically significant (*p* < 0.05), including profiles 10, 11, 12, 13, 14 and 15 (**Figure 4**). Profiles 12, 13 and 15 had the same continuous upregulation patterns (**Figures 5A–C**) and were selected for further analysis, which mostly related to the cell cycle (**Figures 5D, E**), DNA replication (**Figure 5D**), DNA repair (**Figure 5F**) and cell division (**Figures 5D–F**) according to the BP analysis.

### The PPI Network

**Figure 6:**
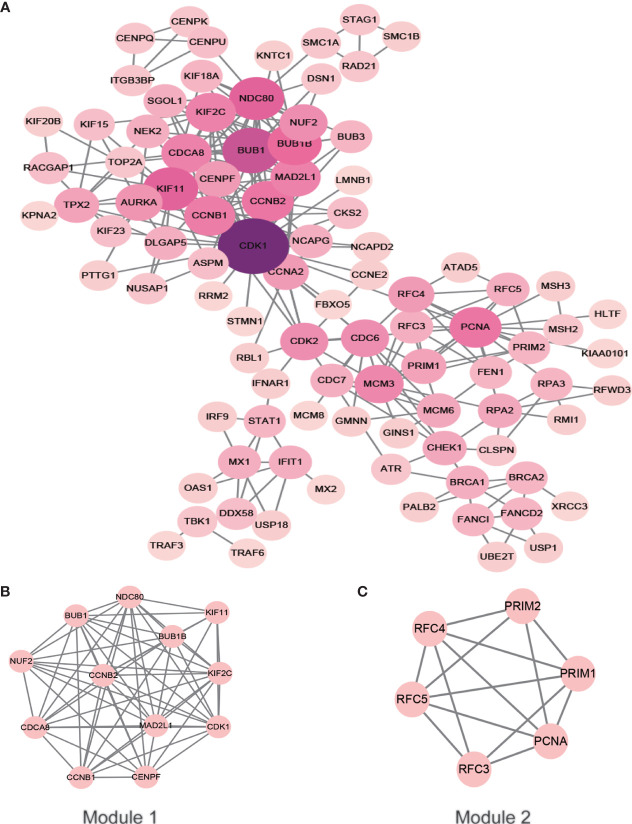
PPI network of the hub genes. The color of the node represents the degree of that node. The edges represent interactions. **(A)** PPI analysis and hub gene screening of target genes. **(B, C)** Top two modules with the highest score (Modules 1 and 2). PPI, protein-protein interactions.

After removing the unconnected nodes and nodes that could not connect to the main network, a PPI network consisting of 267 interactions among 95 nodes was constructed (**Figure 6A**). The top ten genes with the highest degree of connectivity, namely, CDK1, BUB1, KIF11, NDC80, BUB1B, CCNB2, PCNA, CCNB1, MAD2L1 and CDCA8, were selected as hub genes. MCODE in Cytoscape was applied to identify hub modules in the PPI network, which revealed the biological functions of the key protein complexes with the highest degree of connectivity in HSIL and CSCC tissues. In addition, **Figures 6B, C** illustrate that the top two modules with the highest score were selected as potential hub modules (Modules 1 and 2), where hub genes such as CDK1, KIF11, CCNB2, PCNA and CCNB1 were included.

### The ceRNA Network

**Figure 7:**
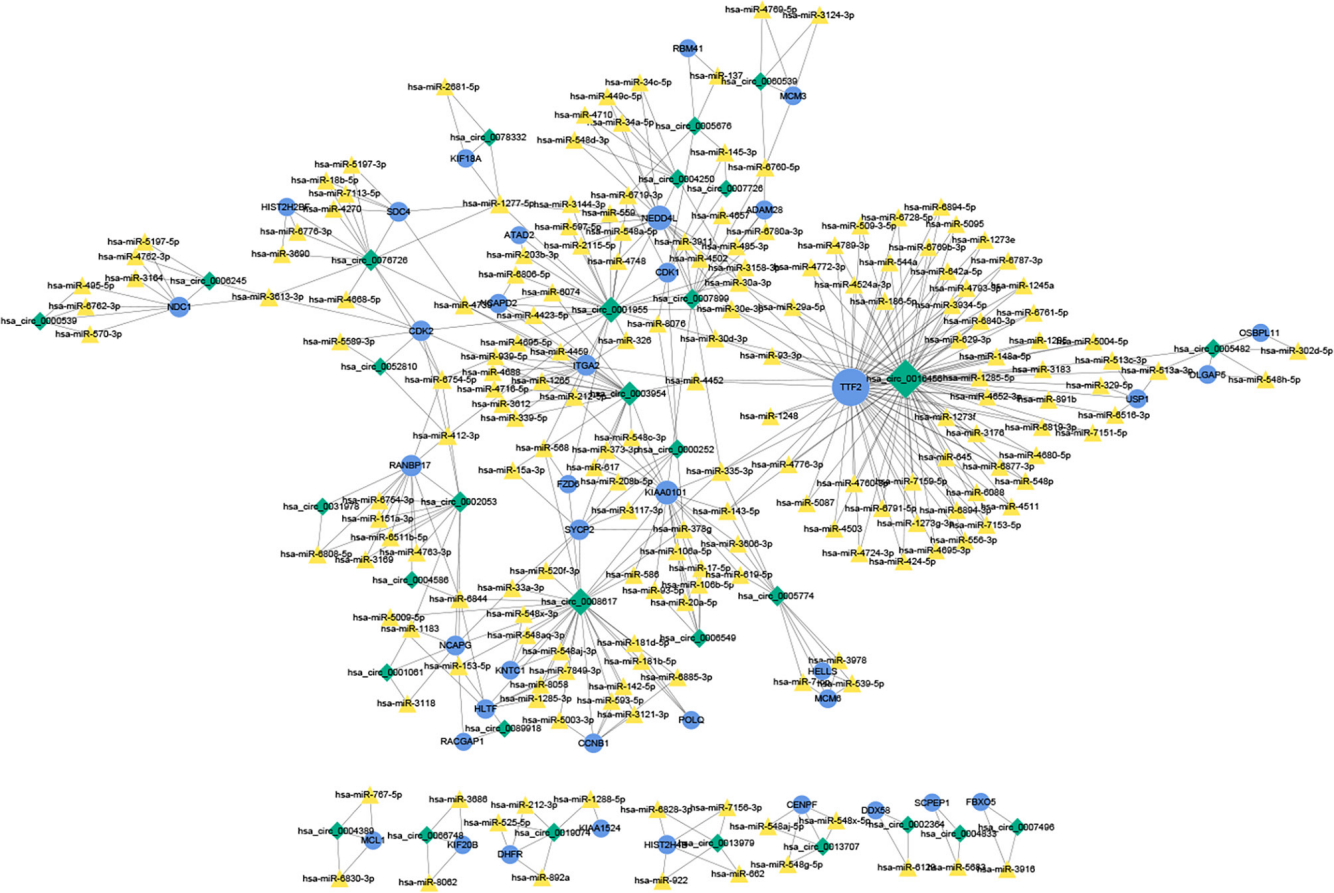
The ceRNA network of circRNAs, miRNAs and mRNAs. The ceRNA relationship pairs were obtained, including 31 circRNAs, 184 miRNAs, and 38 mRNAs. The blue circles represent protein-coding mRNAs, the yellow triangles represent miRNAs and green rhombuses represent circRNAs. The gray solid lines represent the circRNA-miRNA regulatory relationships. The size of the node represents its degree in the network, and the larger the node is, the higher the degree. ceRNA, competing endogenous RNA.

A total of 184 target miRNAs for DECs and DEMs were obtained from the miRanda and TargetScan databases. We constructed a ceRNA network to further investigate the role of mRNAs and circRNAs in cervical carcinogenesis. As shown in **Figure 7**, a total of 479 interactions between the selected genes were identified and visualized. Multiple circRNAs could act as ceRNAs to capture downstream miRNAs, thus influencing the phenotype by regulating mRNAs. Furthermore, hsa-miR-1277-5p, hsa-miR-335-3p, hsa-miR-153-5p, hsa-miR-30a-3p and hsa-miR-412-3p were identified as the miRNAs with the highest degree in the network.

### qRT-PCR and CSCD Analysis

**Figure 8:**
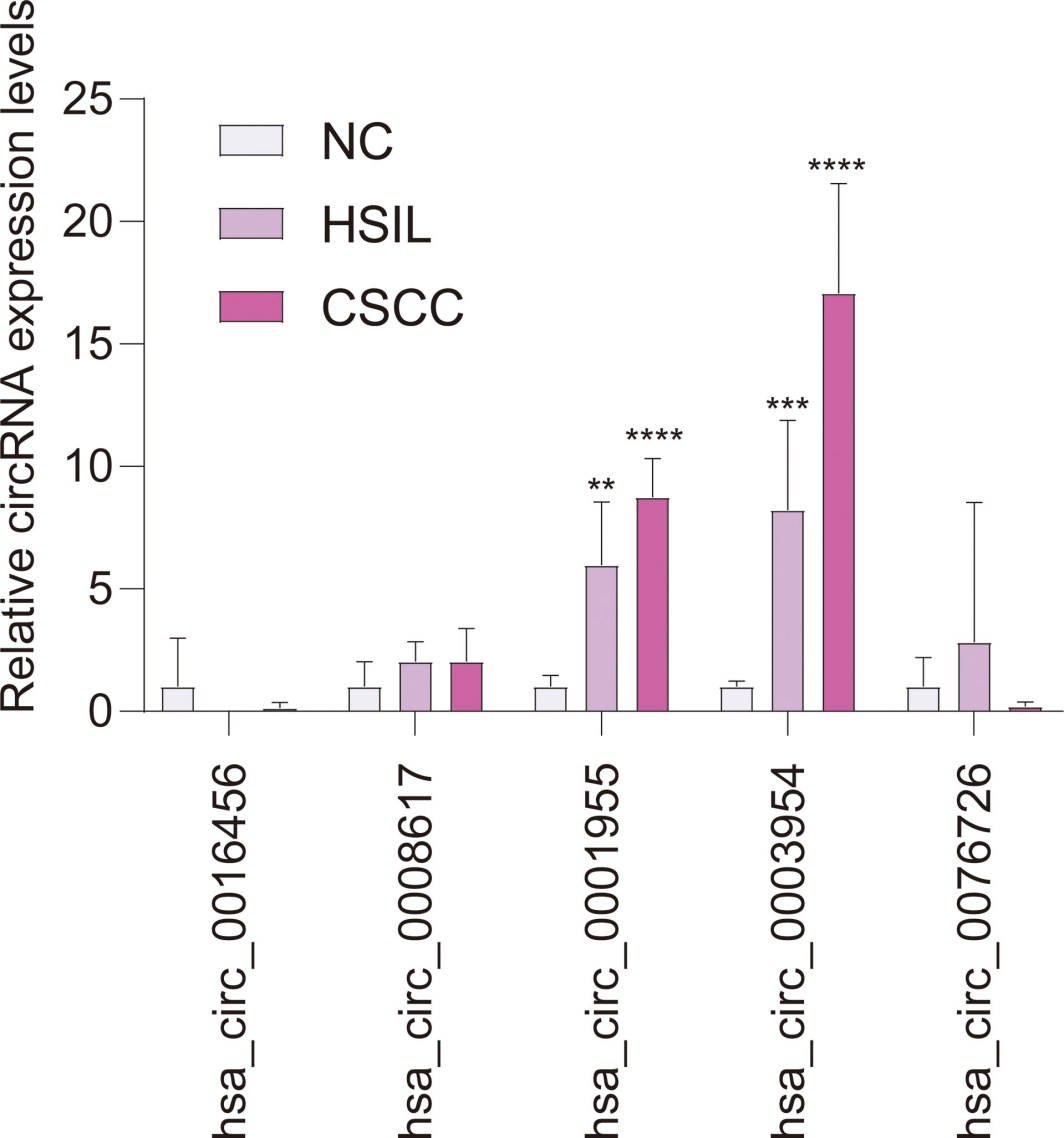
qRT-PCR results of 5 circRNAs in clinical specimens. For qRT-PCR, target gene expression was normalized to GAPDH expression (ΔCt). The results are presented as the mean ± SD (NC-qRT-PCR, n = 5; HSIL-qRT-PCR, n = 5; CSCC-qRT-PCR, n = 5). **p < 0.01, ***p < 0.001, ****p < 0.0001. SD, standard deviation.

**Figure 9:**
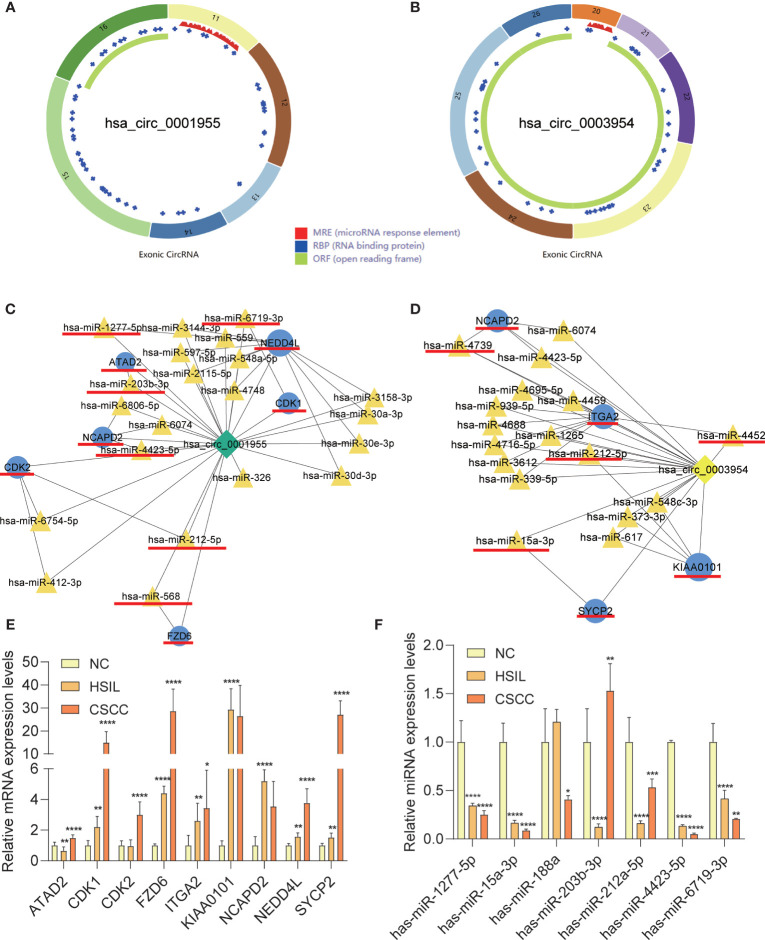
Characteristics of the two circRNAs as determined by the Cancer-specific Circular RNAs database (CSCD) and qRT-PCR results of mRNAs and miRNAs in clinical specimens. **(A)** The structural pattern of hsa_circ_0001955. **(B)** The structural pattern of hsa_circ_0003954 (MRE in red, RBP in blue, ORF in green). **(C)** Hsa_circ_0001955-targeting mRNAs and miRNA. **(D)** Hsa_circ_0003954-targeting mRNAs and miRNA. The mRNAs and miRNAs to be validated are outlined with red lines. **(E)** qRT-PCR results of 9 mRNAs in clinical specimens. **(F)** qRT-PCR results of 10 miRNAs in clinical specimens (hsa-miR-4739, hsa-miR-4452 and hsa-miR-568 were not detected). MRE, microRNA response element; RBP, RNA binding protein; ORF: open reading frame. For qRT-PCR, target mRNAs expression was normalized to GAPDH expression (ΔCt) and miRNAs expression was normalized to U6 expression (ΔCt). The results are presented as the mean ± SD (NC-qRT-PCR, n=5; HSIL-qRT-PCR, n=5; CSCC-qRT-PCR, n=5). *p < 0.05, **p < 0.01, ***p < 0.001, ****p < 0.0001.

qRT-PCR analysis of CSCC, HSIL and NC samples was performed to validate the top 5 circRNAs, namely, hsa_circ_0016456 (degree=64), hsa_circ_0008617 (degree=30), hsa_circ_0001955 (degree=26), hsa_circ_0003954 (degree=21) and hsa_circ_0076726 (degree=14) (**Table 1**). The expression patterns of hsa_circ_0001955 and hsa_circ_0003954 were both continuously upregulated in NC to HSIL and CSCC by qRT-PCR (*p *< 0.01), which was consistent with the above microarray analysis results (**Figure 8**). Moreover, the top 5 miRNAs and mRNAs predicted by ceRNA analysis for hsa_circ_0001955 and hsa_circ_0003954 (**Figures 9C, D** **)** were also validated by qRT-PCR (**Figures 9E, F** **)**. The expression patterns of hsa_circ_0001955/hsa-miR-6719-3p/CDK1, hsa_circ_0001955/hsa-miR-1277-5p/NEDD4L and hsa_circ_0003954/hsa-miR-15a-3p/SYCP2 fit with ceRNA network by qRT-PCR (*p *< 0.01). The structures of hsa_circ_0001955 and hsa_circ_0003954 are presented (**Figures 9A, B** **)** based on the data from CSCD, which indicated that both circRNAs contained MREs.

**Table 1:** Basic characteristics of the top 5 differentially expressed circRNAs in the ceRNA network.

CircRNA ID	Position	Genomic length	Strand	Best transcript	Gene symbol	Regulation
hsa_circ_0016456	chr1:214795421-214830752	35331	+	NM_016343	CENPF	UP
hsa_circ_0008617	chr15:32926133-32928609	2476	+	NM_199357	ARHGAP11A	UP
hsa_circ_0001955	chr15:64495280-64508912	13632	–	NM_022048	CSNK1G1	UP
hsa_circ_0003954	chr18:55983213-56001124	17911	+	NM_001144967	NEDD4L	UP
hsa_circ_0076726	chr6:52128811-52129584	773	–	NM_002388	MCM3	UP

### Silencing of hsa_circ_0003954 Effects Cell Proliferation Progression and Cell Cycle in SiHa Cells

**Figure 10:**
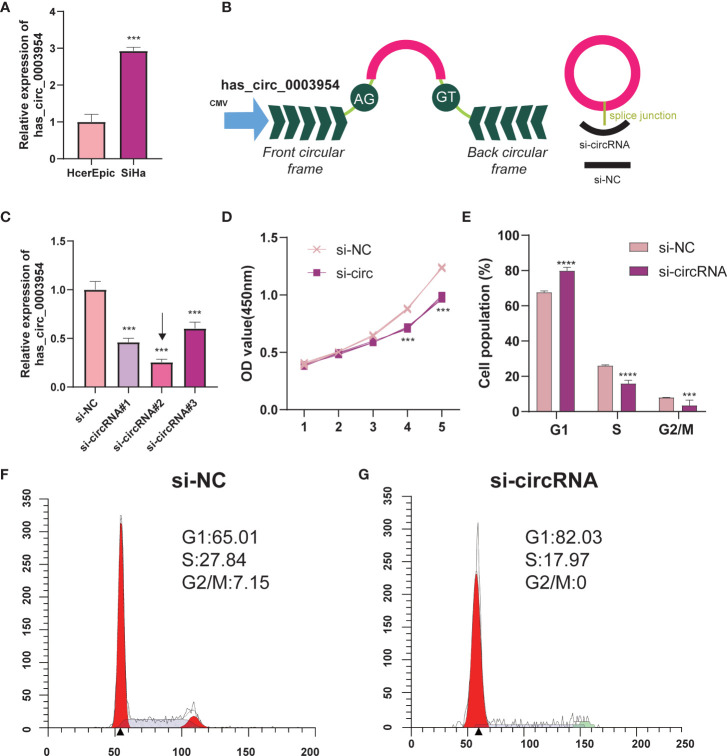
Silencing of hsa_circ_0003954 effects cell proliferation progression and cell cycle in SiHa cells. **(A)** Relative expression of hsa_circ_0003954 in cell lines was determined by qRT-PCR. **(B)** The schematic illustration of hsa_circ_0003954 expression vector and siRNAs. **(C)** qRT-PCR analysis of hsa_circ_0003954 expression in SiHa cells transfected with si-circ or si-NC. **(D)** The growth curves of cells transfected with si-circ or si-NC were evaluated by CCK-8 assays. **(E–G)** The cell cycle progression was analyzed by flow cytometry after transfected with si-circ or si-NC. ***p < 0.001, ****p < 0.0001.

Hsa_circ_0003954, the top upregulated circRNA validated by qRT-PCR, was selected as the prospective circRNA for further research. The expression of hsa_circ_0003954 was evaluated by qRT-PCR in SiHa and HcerEpic cell lines, and the results showed that hsa_circ_0003954 expression was higher in SiHa cells than in HcerEpic cells (**Figure 10A**). Three siRNAs targeting the junction sites of hsa_circ_0003954 (**Figure 10B**) were designed and the qRT-PCR results showed that the expression of hsa_circ_0003954 was significantly downregulated in SiHa cells transfected with the siRNA segments (**Figure 10C**). Of the three siRNAs, si-circRNA#2 was selected for further investigation with the highest silencing effectiveness in SiHa cells.

In the proliferation assay (**Figure 10D**), growth curves performed by CCK-8 assays demonstrated that silencing hsa_circ_0003954 significantly inhibited the proliferation viability of SiHa cells (*p* < 0.001). The cell cycle analysis showed that more SiHa cells were distributed in G1 phase and less in S phase after silencing hsa_circ_0003954, which suggested that SiHa cells were arrested at G1 phase by silencing hsa_circ_0003954 (**Figures 10E–G**).

## Discussion

CircRNAs, playing in a variety of human diseases, contributes to the pathogenesis of abundant cancers due to dysregulated expression, including CSCC (27–34). Nevertheless, because the dynamics of gene expression are characterized by a phasic pattern and cervical carcinogenesis is a gradual process (35), the role of circRNAs in the development of CSCC cannot be fully characterized.

In the present study, we provided comprehensive profiling of the transcriptome involving circRNA and mRNA from NC to HSIL and CSCC. What’s more, we identified 3 mRNA profiles and 3 circRNA profiles that were expressed in a time-dependent manner during the carcinogenic process of the cervix. Interestingly, we observed a significant enrichment of cancer-related signaling pathways in the DEMs over time, such as DNA replication, cell cycle, and DNA repair-related GO functions, based on the DEMs obtained by STEM. Followed by a PPI network (**Figure 6**), CDK1, BUB1, KIF11, NDC80, BUB1B, CCNB2, PCNA, CCNB1, MAD2L1 and CDCA8 were found to be hub genes with an extremely high degree of intramodular connectivity. Among these hub genes, CDK1, KIF11, CCNB2, PCNA and CCNB1 were found in the two hub modules identified by MCODE. Previous studies have identified three genes, CDK1, PCNA and CCNB1 that contribute to the occurrence and development of CSCC (36, 37). However, there is no evidence linking BUB1 and KIF11 with CSCC or of their association with circRNAs. Overall, these hub genes are relevant to carcinogenesis, and further study of their role in CSCC is warranted.

Further alignments and prediction of the selected DECs and DEMs led to the establishment of a ceRNA regulatory network (**Figure 7**). Notably, the expression patterns of hsa_circ_0001955/hsa-miR-6719-3p/CDK1, hsa_circ_0001955/hsa-miR-1277-5p/NEDD4L and hsa_circ_0003954/hsa-miR-15a-3p/SYCP2 fit with ceRNA network by qRT-PCR. It has been found that hsa_circ_0001955/miR-145-5p is the key axis in the carcinogenesis of colorectal cancer (38) and *in vitro*, hsa_circ_0001955 promotes hepatocellular carcinoma cell proliferation, migration, and invasion *via* miR-145-5p/NRAS (39). However, little is known about the role of hsa_circ_0001955 and hsa_circ_0003954 in CSCC. CDK1, target genes of hsa_circ_0001955, have been reported to participate in cervical cancer proliferation, invasion and migration and have been associated with tumor stage and lymph node status (40). Additionally, SYCP2, another important gene identified in our analysis, may contribute to HPV-associated cancer development, according to recent research (41). However, no studies have found that NEDD4L plays an essential regulatory role in CSCC. It has been reported that miR-15a-3p increased radiosensitivity in cervical cancer by targeting TPD52, suggesting that miR-15a-3p may be a potential therapeutic target (42). Little is known about how miR-6719-3p and hsa-miR-1277-5p influence the development and progression of cervical cancer.

To determine the roles of circRNA in the carcinogenesis of cervix, molecular biological experiments are necessary, since our results were based on computational prediction. According to the biological functions and potential mechanisms in cervical carcinogenesis, DNA replication and cell cycle play important roles in CSCC. Therefore, we performed related experiments and found that knocking down hsa_circ_0003954, the top upregulated circRNA, inhibits SiHa cell proliferation and modulates the cell cycle *in vitro*.

In summary, we characterized the circRNA and mRNA transcriptomes of cervical tissues during cervical carcinogenesis. We used STEM analysis to determine the temporal patterns of circRNA and mRNA in cervical carcinogenesis, and we also identified two extremely highly expressed circRNAs, hsa_circ_0001955 and hsa_circ_0003954, that play a potential role in cervical carcinogenesis. Overall, the results of our study shed light on the molecular mechanisms, providing new evidence and insights of CSCC.

## Data Availability Statement

The datasets presented in this study can be found in online repositories. The names of the repository/repositories and accession number(s) can be found below: https://www.ncbi.nlm.nih.gov/, GSE166466.

## Ethics Statement

The studies involving human participants were reviewed and approved by Ethics Committee of Second Hospital of Shanxi Medical University. The patients/participants provided their written informed consent to participate in this study.

## Author Contributions

WW designed and supervised this project. HL, YL, and WW analyzed the data, and wrote the manuscript. HL and YL performed the experiments, YZ, JC, XZ, BZ, LG, and WW revised the manuscript. HL and YL contributed to data interpretation. All authors contributed to the article and approved the submitted version.

## Funding

This work was supported by the grant from the Key Research and Development Program of Shanxi (201903D321152).

## Conflict of Interest

The authors declare that the research was conducted in the absence of any commercial or financial relationships that could be construed as a potential conflict of interest.

## Publisher’s Note

All claims expressed in this article are solely those of the authors and do not necessarily represent those of their affiliated organizations, or those of the publisher, the editors and the reviewers. Any product that may be evaluated in this article, or claim that may be made by its manufacturer, is not guaranteed or endorsed by the publisher.

## References

[1] Sung H Ferlay J Siegel RL Laversanne M Soerjomataram I Jemal A. Global Cancer Statistics 2020: GLOBOCAN Estimates of Incidence and Mortality Worldwide for 36 Cancers in 185 Countries. CA: Cancer J Clin (2021) 71(3):209–49. 10.3322/caac.21660 33538338

[2] Zhao W Liu Y Zhang L Ding L Li Y Zhang H. MicroRNA-154-5p Regulates the HPV16 E7-pRb Pathway in Cervical Carcinogenesis by Targeting CUL2. J Cancer (2020) 11: (18):5379–89. 10.7150/jca.45871 PMC7391205 32742484

[3] Lin Y-H Hsiao Y-H Wu W-J Yang S-F Hsu C-F Kang Y-T. Relationship of Genetic Variant Distributions of WW Domain-Containing Oxidoreductase Gene With Uterine Cervical Cancer. Int J Med Sci (2018) 15(10):1005–13. 10.7150/ijms.25553 PMC6036151 30013442

[4] Tainio K Athanasiou A Tikkinen KAO Aaltonen R Cárdenas J Hernándes. Clinical Course of Untreated Cervical Intraepithelial Neoplasia Grade 2 Under Active Surveillance: Systematic Review and Meta-Analysis. BMJ (Clin Res ed) (2018) 360:k499. 10.1136/bmj.k499 PMC5826010 29487049

[5] Feng D Yan K Liang H Liang J Wang W Yu H. CBP-Mediated Wnt3a/β-Catenin Signaling Promotes Cervical Oncogenesis Initiated by Piwil2. Neoplasia (2021) 23(1):1–11. 10.1016/j.neo.2020.10.013 33190089 PMC7674161

[6] Liu J Liu T Wang X He A. Circles Reshaping the RNA World: From Waste to Treasure. Mol Cancer (2017) 16:58. 10.1186/s12943-017-0630-y 28279183 PMC5345220

[7] Li J Yang J Zhou P Le Y Gong ZJAJCR. Circular RNAs in Cancer: Novel Insights Into Origins, Properties, Functions and Implications. Am J Cancer Res (2015) 5(2):472–80. PMC4396047 25973291

[8] Chen Y Li C Tan C Liu X. Circular RNAs: A New Frontier in the Study of Human Diseases. J Med Genet (2016) 53(6):359–65. 10.1136/jmedgenet-2016-103758 26945092

[9] Szabo L Salzman J. Detecting Circular RNAs: Bioinformatic and Experimental Challenges. Nat Rev Genet (2016) 17(11):679–92. 10.1038/nrg.2016.114 PMC5565156 27739534

[10] Xuan L Qu L Zhou H Wang P Yu H Wu T. Circular RNA: A Novel Biomarker for Progressive Laryngeal Cancer. Am J Trans Res (2016) 8(2):932–9. PMC4846937 27158380

[11] Zheng H Chen T Li C Xu C Ding C Chen J. A Circular RNA Hsa_Circ_0079929 Inhibits Tumor Growth in Hepatocellular Carcinoma. Cancer Manage Res (2019) 11:443–54. 10.2147/cmar.S189338 PMC6322497 30655696

[12] Hao S Cong L Qu R Liu R Zhang G Li Y. Emerging Roles of Circular RNAs in Colorectal Cancer. OncoTargets Ther (2019) 12:4765–77. 10.2147/ott.S208235 PMC6590902 31354303

[13] Wu W Zhen T Yu J Yang Q. Circular RNAs as New Regulators in Gastric Cancer: Diagnosis and Cancer Therapy. Front Oncol (2020) 10:1526. 10.3389/fonc.2020.01526 33072546 PMC7531269

[14] Song X Zhang N Han P Moon B Lai R Wang K. Circular RNA Profile in Gliomas Revealed by Identification Tool UROBORUS. Nucleic Acids Res (2016) 44(9):e87. 10.1093/nar/gkw075 26873924 PMC4872085

[15] Cao Y Li J Jia Y Zhang R Shi H. GNB1CircRNA Circ_POLA2 Promotes Cervical Squamous Cell Carcinoma Progression *via* Regulating miR-326. Front Oncol (2020) 10:959. 10.3389/fonc.2020.00959 32766125 PMC7381119

[16] Tornesello ML Faraonio R Buonaguro L Annunziata C Starita N Cerasuolo A. The Role of microRNAs, Long Non-Coding RNAs, and Circular RNAs in Cervical Cancer. Front Oncol (2020) 10:150. 10.3389/fonc.2020.00150 32154165 PMC7044410

[17] Zhou Y Shen L Wang Y Zhou CJN. The Potential of ciRS-7 for Predicting Onset and Prognosis of Cervical Cancer. Neoplasma (2020) 67(2):312–22. 10.4149/neo_2019_190415N334 31884800

[18] Zhao J Lee EE Kim J Yang R Chamseddin B Ni C. Transforming Activity of an Oncoprotein-Encoding Circular RNA From Human Papillomavirus. Nat Commun (2019) 10(1):2300. 10.1038/s41467-019-10246-5 31127091 PMC6534539

[19] Bhatla N Berek J Cuello Fredes M Denny L Grenman S Karunaratne K. Revised FIGO Staging for Carcinoma of the Cervix Uteri. Int J Gynaecol Obstet (2019) 145(1):129–35. 10.1002/ijgo.12749 30656645

[20] Zhou Y Zhou B Pache L Chang M Khodabakhshi AH Tanaseichuk O. Metascape Provides a Biologist-Oriented Resource for the Analysis of Systems-Level Datasets. Nat Commun (2019) 10(1):1523. 10.1038/s41467-019-09234-6 30944313 PMC6447622

[21] Harris M Clark J Ireland A Lomax J Ashburner M Foulger R. The Gene Ontology (GO) Database and Informatics Resource. Nucleic Acids Res (2004) 32:D258–61. 10.1093/nar/gkh036 PMC308770 14681407

[22] Ernst J Bar-Joseph Z. STEM: A Tool for the Analysis of Short Time Series Gene Expression Data. BMC Bioinf (2006) 7:191. 10.1186/1471-2105-7-191 PMC1456994 16597342

[23] Szklarczyk D Morris JH Cook H Kuhn M Wyder S Simonovic M. The STRING Database in 2017: Quality-Controlled Protein-Protein Association Networks, Made Broadly Accessible. Nucleic Acids Res (2017) 45(D1):D362–d8. 10.1093/nar/gkw937 PMC5210637 27924014

[24] Enright A John B Gaul U Tuschl T Sander C Marks D. MicroRNA Targets in Drosophila. Genome Biol (2003) 5(1):R1. 10.1186/gb-2003-5-1-r1 14709173 PMC395733

[25] Agarwal V Bell G Nam J Bartel D. Predicting Effective microRNA Target Sites in Mammalian mRNAs. eLife (2015) 4:e05005. 10.7554/eLife.05005 PMC4532895 26267216

[26] Xia S Feng J Chen K Ma Y Gong J Cai F. CSCD: A Database for Cancer-Specific Circular RNAs. Nucleic Acids Res (2018) 46(D1):D925–d9. 10.1093/nar/gkx863 PMC5753219 29036403

[27] Du WW Chao Z Yang W Yong T Mehwish AF Yang BBJT. Identifying and Characterizing circRNA-Protein Interaction. Theranostics (2017) 7(17):4183–91. 10.7150/thno.21299 PMC5695005 29158818

[28] Ebbesen KK Hansen TB Kjems JRJRB. Insights Into Circular RNA Biology. RNA Biol (2017) 14(8):1035–45. 10.1080/15476286.2016.1271524 PMC5680708 27982727

[29] Pamudurti NR Bartok O Jens M Ashwal-Fluss R Stottmeister C Ruhe L. Translation of CircRNAs. Mol Cell (2017) 66(1). 10.1016/j.molcel.2017.02.021 PMC5387669 28344080

[30] Rong D Sun H Li Z Liu S Cao HJO. An Emerging Function of circRNA-miRNAs-mRNA Axis in Human Diseases. Oncotarget (2015) 8(42):73271–81. 10.18632/oncotarget.19154 PMC5641211 29069868

[31] Gong J Jiang H Shu C Hu MQ Huang Y Liu Q. Integrated Analysis of Circular RNA-Associated ceRNA Network in Cervical Cancer: Observational Study. Medicine (2019) 98(34):e16922. 10.1097/md.0000000000016922 31441876 PMC6716739

[32] Huang H Chen YF Du X Zhang C. Identification and Characterization of Tumorigenic Circular RNAs in Cervical Cancer. Cell Signal (2020) 73:109669. 10.1016/j.cellsig.2020.109669 32423867

[33] Li S Teng S Xu J Su G Zhang Y Zhao J. Microarray is an Efficient Tool for circRNA Profiling. Briefings Bioinf (2019) 20(4):1420–33. 10.1093/bib/bby006 29415187

[34] Yi Y Liu Y Wu W Wu K Zhang W. Reconstruction and Analysis of circRNA−miRNA−mRNA Network in the Pathology of Cervical Cancer. Oncol Rep (2019) 41(4):2209–25. 10.3892/or.2019.7028 PMC6412533 30816541

[35] Sun YH Chou YH Ou CC Ng SC Shen HP Lee YC. Investigation of Metastasis-Associated in Colon Cancer-1 Genetic Variants in the Development and Clinicopathologcial Characteristics of Uterine Cervical Cancer in Taiwanese Women. Int J Med Sci (2020) 17(4):490–7. 10.7150/ijms.40204 PMC7053312 32174779

[36] Branca M Ciotti M Giorgi C Santini D Di Bonito L Costa S. Up-Regulation of Proliferating Cell Nuclear Antigen (PCNA) Is Closely Associated With High-Risk Human Papillomavirus (HPV) and Progression of Cervical Intraepithelial Neoplasia (CIN), But Does Not Predict Disease Outcome in Cervical Cancer. Eur J Obstet Gynecol Reprod Biol (2007) 130(2):223–31. 10.1016/j.ejogrb.2006.10.007 17098349

[37] Li S Liu N Piao J Meng F Li Y. CCNB1 Expedites the Progression of Cervical Squamous Cell Carcinoma *via* the Regulation by FOXM1. OncoTargets Ther (2020) 13:12383–95. 10.2147/OTT.S279951 PMC7721124 33299327

[38] Ding B Yao M Fan W Lou W. Whole-Transcriptome Analysis Reveals a Potential Hsa_Circ_0001955/Hsa_Circ_0000977-Mediated miRNA-mRNA Regulatory Sub-Network in Colorectal Cancer. Aging (2020) 12(6):5259–79. 10.18632/aging.102945 PMC7138558 32221048

[39] Ding B Fan W Lou W. *In Vitro* hsa_circ_0001955 Enhances Proliferation, Migration, and Invasion of HCC Cells Through miR-145-5p/NRAS Axis. Mol Ther Nucleic Acids (2020) 22:445–55. 10.1016/j.omtn.2020.09.007 PMC7554323 33230448

[40] Luo Y Wu Y Peng Y Liu X Bie J Li S. Systematic Analysis to Identify a Key Role of CDK1 in Mediating Gene Interaction Networks in Cervical Cancer Development. Irish J Med Sci (2016) 185(1):231–9. 10.1007/s11845-015-1283-8 25786624

[41] Masterson L Sorgeloos F Winder D Lechner M Marker A Malhotra S. Deregulation of SYCP2 Predicts Early Stage Human Papillomavirus-Positive Oropharyngeal Carcinoma: A Prospective Whole Transcriptome Analysis. Cancer Sci (2015) 106(11):1568–75. 10.1111/cas.12809 PMC4714680 26334652

[42] Wu Y Huang J Xu H Gong Z. Over-Expression of miR-15a-3p Enhances the Radiosensitivity of Cervical Cancer by Targeting Tumor Protein D52. BioMed Pharmacother (2018) 105:1325–34. 10.1016/j.biopha.2018.06.033 30021370

