# Supplementary material for: Comprehensive Analysis of circRNA Expression Profiles During Cervical Carcinogenesis
Source: Front Oncol. 2021 Aug 31;11:676609. doi: 10.3389/fonc.2021.676609 (PMC8438239; doi:10.3389/fonc.2021.676609)
Supplement: Supplementary file 1 [file DataSheet_1.docx]

**
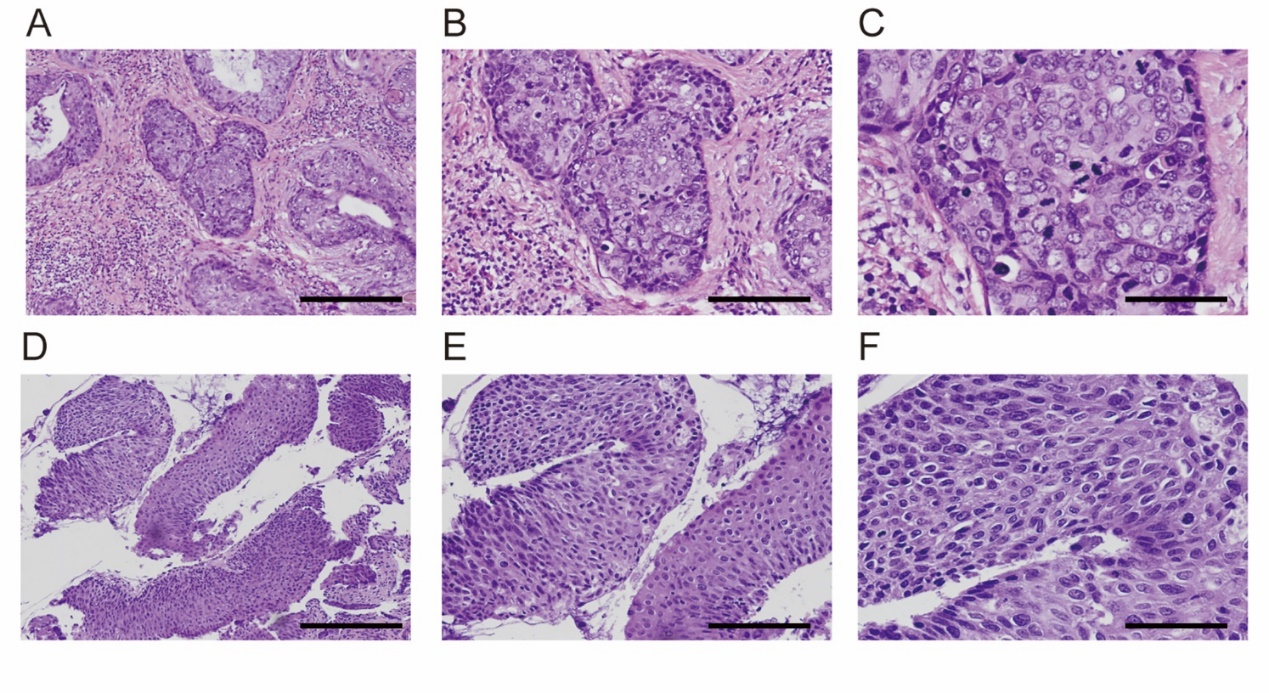
Figure S1** Histology of cervical specimens from CSCC (A-C) and HSIL (D-F) patients. Paraffin-embedded cervical specimens were stained with hematoxylin and eosin (H&E). Scale bar = 200 µm (A, D), 100 µm (B, E) and 50 µm (C, F).

**Table S1** Baseline characteristics of the included individuals.

| Group | Age | HPV | TCT | FIGO Stages | Cause(operation) | Usage |
| --- | --- | --- | --- | --- | --- | --- |
| NC | 43 | 16 | NILM | - | Colposcopy Biopsy | Microarray |
|  | 36 | 16 | NILM | - | Colposcopy Biopsy | Microarray |
|  | 60 | 16 | NILM | - | Colposcopy Biopsy | Microarray |
|  | 57 | 16 | NILM | - | Colposcopy Biopsy | Microarray |
|  | 38 | 16 | NILM | - | Colposcopy Biopsy | Microarray |
|  | 35 | 16 | NILM | - | Colposcopy Biopsy | Microarray |
|  | 40 | 16 | NILM | - | Colposcopy Biopsy | Microarray |
|  | 63 | 16 | ASC | - | Colposcopy Biopsy | qRT-PCR |
|  | 47 | 16 | ASC | - | Colposcopy Biopsy | qRT-PCR |
|  | 46 | 16 | ASC | - | Colposcopy Biopsy | qRT-PCR |
|  | 34 | 16 | ASC | - | Colposcopy Biopsy | qRT-PCR |
|  | 32 | 16 | ASC | - | Colposcopy Biopsy | qRT-PCR |
| HSIL | 25 | 16 | LSIL | - | Colposcopy Biopsy | Microarray |
|  | 43 | 16 | ASC | - | Colposcopy Biopsy | Microarray |
|  | 27 | 16 | ASC | - | Colposcopy Biopsy | Microarray |
|  | 46 | 16 | ASC | - | Cervical Conization | Microarray |
|  | 33 | 16 | LSIL | - | Colposcopy Biopsy | Microarray |
|  | 39 | 16 | ASC | - | Cervical Conization | Microarray |
|  | 38 | 16 | LSIL | - | Colposcopy Biopsy | qRT-PCR |
|  | 32 | 16 | ASC | - | Colposcopy Biopsy | qRT-PCR |
|  | 45 | 16 | HSIL | - | Cervical Conization | qRT-PCR |
|  | 42 | 16 | ASC | - | Colposcopy Biopsy | qRT-PCR |
|  | 25 | 16 | NILM | - | Colposcopy Biopsy | qRT-PCR |
| CSCC | 61 | 16 | HSIL | IB1 | Hysterectomy | Microarray |
|  | 54 | 16 | LSIL | IIA1 | Hysterectomy | Microarray |
|  | 49 | 16 | HSIL | IB1 | Hysterectomy | Microarray |
|  | 58 | 16 | ASC | IB1 | Hysterectomy | Microarray |
|  | 48 | 16 | HSIL | IA2 | Hysterectomy | Microarray |
|  | 54 | 16 | NILM | IB1 | Hysterectomy | Microarray |
|  | 60 | 16 | HSIL | IB1 | Hysterectomy | Microarray |
|  | 62 | 16 | HSIL | IB1 | Hysterectomy | qRT-PCR |
|  | 58 | 16 | HSIL | IB1 | Hysterectomy | qRT-PCR |
|  | 56 | 16 | LSIL | IA1 | Hysterectomy | qRT-PCR |
|  | 53 | 16 | NILM | IA1 | Hysterectomy | qRT-PCR |
|  | 51 | 16 | NILM | IB1 | Hysterectomy | qRT-PCR |

TCT, Thin Prep cytologic test; NILM, negative for intraepithelial lesion or malignancy; ASC, atypical squamous cell; LSIL, low-grade squamous intraepithelial lesion; HSIL, high-grade squamous intraepithelial lesion; CSCC, cervical squamous cell carcinoma.

**Table S2** Sequences of primers used in qRT-PCR.

| Gene | Forward primer (5'–3') | Reverse primer (5'–3') |
| --- | --- | --- |
| GAPDH | GGGCATCCTGGGCTACACTG | AAATGAGCTTGACAAAGTGGTCG |
| hsa_circ_0016456 | CTGAGTTTGAGCCAGAGGGAC | GCAGGCGTAGTTCCAACTCAT |
| hsa_circ_0008617 | TACCGGATGTCTTGGACAGG | GCAGCCTGCTTTTCCTGATT |
| hsa_circ_0001955 | ACAGCAGCCTCTTCGAAATCA | CTCCAAGCTAGGGCCAAGGA |
| hsa_circ_0003954 | GAACAGGGCCATCTACCACC | GTCGCTCCATGGTTGGATCT |
| hsa_circ_0076726  U6  hsa-miR-212-5p  hsa-miR-568  hsa-miR-6719-3p  hsa-miR-4423-5p  hsa-miR-203b-3p  hsa-miR-1277-5p  hsa-miR-15a-3p  hsa-miR-4739  hsa-miR-188-3p  CDK2  FZD6  CDK1  NCAPD2  ATAD2  NEDD4L  ITGA2  SYCP2  KIAA0101 | AGCTTTCTTTGCAGTAGCACCA  CTCGCTTCGGCAGCACA  CGCGACCTTGGCTCTAGACTG  GCGCGCGATGTATAAATGTAT  GCGCGTCTGACATCAGTGATT  CGCGAGTTGCCTTTTTGTTC  GCGCGTTGAACTGTTAAGAACC  GCGCGCGAAATATATATATATATGT  GCGCAGGCCATATTGTGCT  GGGAGGAGGAGCGGAGG  CGCTCCCACATGCAGGG  ATGGATGCCTCTGCTCTCACTG  GGCAGTGTATCTGAAAGTGCGC  TCAGTCTTCAGGATGTGCTTATG  TGGAGGGGTGAATCAGTATGT  AAGGAAGTTGAAACCTACCACCG  CCCTGCACCCCTGTACTTAAGAG  GGGAATCAGTATTACACAACGGG  ACCAGATTCACAGGCAGCGGAA  GAGATTTGCTTAGATTGTTGTACTG | CAAGAGGGCCACCTTGAATG  AACGCTTCACGAATTTGCGT  AGTGCAGGGTCCGAGGTATT  AGTGCAGGGTCCGAGGTATT  AGTGCAGGGTCCGAGGTATT  AGTGCAGGGTCCGAGGTATT  AGTGCAGGGTCCGAGGTATT  AGTGCAGGGTCCGAGGTATT  AGTGCAGGGTCCGAGGTATT  AGTGCAGGGTCCGAGGTATT  AGTGCAGGGTCCGAGGTATT  CCCGATGAGAATGGCAGAAAGC  GATGTGGAACCTTTGAGGCTGC  GTACTGACCAGGAGGGATAGAA  GCGGGATACCACTTTTATCAGG  GCAAGTTGCTCCGTTATTTCCA  TGACACGACTTATCAGGATGTACCC  CCACAACATCTATGAGGGAAGGG  GGTGTCCAACATGCCCATTTGC  GAGTGACAATATGGCACCATTC |

**Table S3** Hsa_circ_0003954 siRNAs sequences and negative control siRNA sequence.

|  | Sequence |
| --- | --- |
| Negative control siRNA | 5′-UUCUCCGAACGUGUCACGUTT-3′ |
| siRNA 1 | 5′-GGCCAUCUACCACCGACACTT-3′ |
| siRNA 2 | 5′-CCACCGACACGAGACGACUTT-3′ |
| siRNA 3 | 5′-CTCTTCGAAATCAGGTGAA-3′ |
